# The Performance of Opioid-Free Anesthesia for Bariatric Surgery in Clinical Practice

**DOI:** 10.1007/s11695-023-06584-5

**Published:** 2023-04-27

**Authors:** Stefan Ulbing, Lukas Infanger, Edith Fleischmann, Gerhard Prager, Thomas Hamp

**Affiliations:** 1 Department of Anaesthesia, Intensive Care Medicine and Pain Medicine, Medical University of Vienna, Währinger Gürtel 18-20, 1090 Vienna, Austria; 2 Ludwig Boltzmann Institute Digital Health and Patient Safety, Medical University of Vienna, Spitalgasse 23, 1090 Vienna, Austria; 3 Division of Visceral Surgery, Department of General Surgery, Medical University of Vienna, Währinger Gürtel 18-20, 1090 Vienna, Austria

**Keywords:** Bariatric surgery, Opioid-free anesthesia, Postoperative pain, Clinical practice

## Abstract

**Purpose:**

Opioid-free anesthesia (OFA) is an alternative to conventional opioid-based anesthesia (OBA) in patients undergoing bariatric surgery. Several small studies and a meta-analysis have suggested advantages of OFA for bariatric surgery, but current evidence is still contradictory, and a universally accepted concept has not yet been established. The purpose of this study was to determine whether patients undergoing bariatric surgery experience less postoperative pain and better postoperative recovery when anesthetized with an OFA regimen than with an OBA regimen.

**Materials and Methods:**

This prospective observational cohort study, conducted between October 2020 and July 2021, compared patients receiving OFA with patients receiving OBA. Patients were visited 24 and 48 h after the surgical procedure and asked about their postoperative pain using the visual analogue scale (VAS). Additionally, the quality of recovery-40 questionnaire (QoR-40) and the postoperative opioid requirements were recorded.

**Results:**

Ninety-nine patients were included and analyzed in this study (OFA: *N* = 50; OBA: *N* = 49). The OFA cohort exhibited less postoperative pain than the OBA cohort within 24 h (VAS median [interquartile range (IQR)]: 2.2 [1–4.4] vs. 4.1 [2–6.5]; *P* ≤ 0.001) and 48 h (VAS median [IQR]: 1.9 [0.4–4.1] vs. 3.1 [1.4–5.8]; *P* ≤ 0.001) postoperatively. Additionally, the OFA cohort had higher QoR-40 scores and required less opioid therapy postoperatively.

**Conclusion:**

Based on our results the use of OFA for bariatric surgery results in less pain, reduced opioid requirements, and improved postoperative recovery—adding additional evidence regarding the use of OFA in everyday clinical practice.

**Graphical Abstract:**

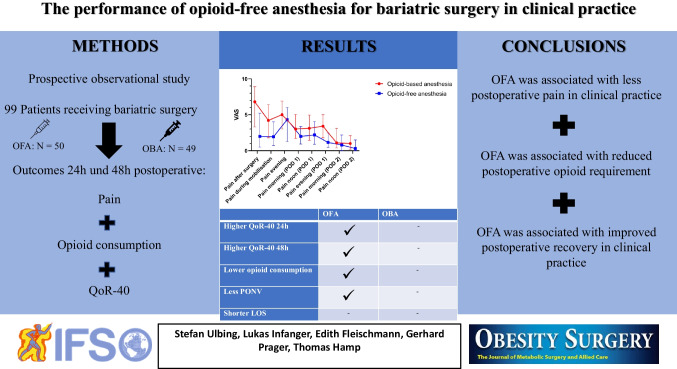

**Supplementary Information:**

The online version contains supplementary material available at 10.1007/s11695-023-06584-5.

## Introduction

Opioid-free anesthesia (OFA) has been used for several years in an attempt to reduce risks and to improve recovery after surgery by decreasing opioid-induced side effects. For OFA, opioids are replaced by anesthetic adjuncts, such as ketamine, lidocaine, dexmedetomidine, and magnesium [1–5]. The use of OFA has been investigated for various types of surgery (e.g. gynecologic surgery [4, 6], bariatric surgery [1], cardiac surgery [7], urological surgery [8], orthopedic surgery [9]), and has been demonstrated to reduce postoperative pain [1, 10, 11] and postoperative nausea and vomiting (PONV) [6, 10, 11]. There is also evidence that OFA improves postoperative recovery [1, 4, 12]. However, with respect to adverse events, there is currently conflicting evidence regarding the safety of the use of OFA. A large prospective, randomized controlled trial had to be stopped early due to safety concerns due to the occurrence of bradycardia [13]. This contradicts two meta-analyses that assessed the evidence grade in this regard as moderate [14] or low [15].

Patients receiving bariatric surgery have a high risk for PONV [16, 17] and postoperative respiratory depression due to a high prevalence of obstructive sleep apnea syndrome in this patient population [18–20], necessitating an investigation of opiate-free anesthesia in this patient cohort [21]. In a recent meta-analysis of several small, randomized trials, Hung et al. demonstrated that the use of OFA in patients undergoing bariatric surgery resulted in a reduction in postoperative pain and PONV but not a reduction in postoperative opioid consumption within 24 h postoperatively [10]. This raises the question of whether these findings can be confirmed in a real-world scenario that is, in everyday clinical practice without a controlled trial setting. Furthermore, only a period of 24 h postoperatively was evaluated; thus, potential differences in postoperative pain over a longer postoperative period remain unclear. No significant difference was found for postoperative recovery (measured by the quality of recovery-40 questionnaire [QoR-40]) [22], but only two studies could be used to analyze this parameter, and in one of them, an additional bilateral oblique subcostal transverse abdominis plane block was performed in both groups [23], which may distort comparability. In a large retrospective analysis, the use of OFA was shown to result in a shorter LOS, indicating better recovery [24].

At our center, an OFA regimen has been used in parallel with an opioid-based regime (OBA) in bariatric surgery since June 2018. This study aimed to investigate the effects of these concepts on postoperative pain and recovery after a period of 48 h in patients who underwent bariatric surgery in everyday clinical practice.

## Materials and Methods

### Study Design

This prospective single-center cohort study examined postoperative pain, perioperative opioid consumption, PONV and the quality of recovery in patients undergoing first-intervention bariatric surgery between October 2020 and July 2021 at the Medical University of Vienna, Austria. We compared patients receiving OFA with patients receiving OBA. Informed consent was obtained from all individual participants included in the study. The study was approved by the Ethics Committee of the Medical University of Vienna (EK 1748/2020) and was performed in accordance with the principles of Good Clinical Practice and the Declaration of Helsinki.

### Inclusion Criteria

The operating room schedule was regularly screened for eligible study participants, and eligible patients were contacted. Patients with a body mass index (BMI) greater than 35 kg/m^2^ undergoing first-time bariatric surgery under general anesthesia were included. Patient age was restricted to 18–65 years, and written informed consent was required before inclusion. Only patients who could be interviewed postoperatively were included. If a postoperative survey was not possible (e.g. study team was unavailable or language barrier), the patient was not included. Patients for whom the anesthetic regimen was changed intraoperatively (from OFA to OBA or vice versa) were excluded. When the sample size was reached in one cohort, no more patients were included in this cohort.

### Cohorts

Patients were assigned to respective cohorts according to the type of anesthesia (OFA/OBA) performed.

### Anesthetic Regimens Used/Treatment

The form of anesthesia (OFA/OBA) used was chosen by the anesthetist in charge and was determined on the basis of the standard operating procedure (SOP) of the respective operating room of the respective area. In our center, there are two sections of operating rooms performing bariatric surgery. In section 1, mainly OFA is applied, in section 2 OBA. Whether a patient is assigned to section 1 or 2 is random. To provide data from everyday clinical practice without a controlled trial setting, the study team had no influence on the treatment overall or choice of anesthesia at any time. OFA was performed according to an SOP. For anesthesia induction, a continuous intravenous infusion of s-ketamine (1.25 mg/ml), dexmedetomidine (10 µg/ml), and lidocaine (10 mg/ml) was started at 20 ml/h (s-ketamine: 25 mg/h; dexmedetomidine: 200 µg/h; lidocaine: 200 mg/h). Then, 200–250 mg propofol and 100 mg rocuronium were administered.

For maintenance, depending on the individual requirements of the patient, the syringe pump was set at 5 to 10 ml/h (s-ketamine: 6.25–12.5 mg/h; dexmedetomidine 50–100 µg/h; lidocaine 50–100 mg/h), and a volatile anesthetic (sevoflurane/desflurane) was administered. In addition, metamizole (2.5 g), magnesium sulfate (2–4 g), and parecoxib (40 mg) or diclofenac (75 mg) were administered intraoperatively. In the recovery room, continuous infusion could be continued at 5 ml/h as first-line analgesic therapy. Piritramide was available as a rescue therapy. This SOP is a modification of Mulier’s OFA protocol [1].

OBA was conducted by administering fentanyl, propofol, and rocuronium for induction and remifentanil in combination with a volatile anesthetic (sevoflurane/desflurane) for maintenance. Piritramide was the first line of analgesic therapy for the treatment of acute postoperative pain.

Detailed descriptions of the anesthetic concepts are provided in the supplementary material.

### Determination of Outcomes

Participants were questioned 24 h and 48 h after surgery to assess pain perception (using the visual analogue scale (VAS)) at different time points and postoperative recovery (using the quality of recovery-40 (QoR-40) questionnaire) during the first 48 h after surgery. In addition, the opioid (piritramide) requirement was recorded 24 h after surgery, based on documentation in the patient files.

### Primary Outcome

The primary endpoint of this study was the difference in the VAS within the first 24 h after surgery between the two cohorts. For this purpose, the participating patients were asked about their pain perception five times within this period: immediately after surgery, during initial mobilization, in the evening after surgery, in the morning on the first postoperative day (POD) day, and at noon on the first POD.

### Secondary Outcomes

For postoperative pain assessment between 24 and 48 h, patients were asked about their pain perception at three additional time points using the VAS score: in the evening on the first POD, in the morning on the second POD, and at noon on the second POD. Postoperative recovery was determined using the QoR-40 questionnaire 24 and 48 h postoperatively. To assess PONV separately, we evaluated the three PONV-specific parameters (nausea, vomiting, dry retching) of the QoR-40 questionnaire at 24 h (PONV-score: 3 = worst, 15 = best) and recorded whether droperidol was administered in the post anesthesia care unit (PACU). Postoperative opioid consumption within 24 h was recorded in the recovery room and in the ward in both cohorts, as well as in the administration of s-ketamine, dexmedetomidine, and lidocaine in the OFA cohort in the recovery room. The length of hospital stay was documented as well as whether patients were discharged within 24 to 48 h postoperatively before the second survey.

### Statistical Analysis

No formal sample size calculation was performed. We planned to observe 50 patients in the OFA group and 50 patients in the OBA group. The number of cases was determined to allow for examining a larger patient collective than had previously been done in smaller randomized studies [2–6]. Normal distribution for continuous data was tested using the Kolmogorov–Smirnov test. Normally distributed data are presented as mean (SD), and non-normally distributed data are presented as median (interquartile range [IQR], 25^th^ to 75^th^ percentile). The main endpoint of this study (VAS within 24 h) was compared between the two groups with a Mann–Whitney U test. For the secondary outcome parameters, exploratory Mann–Whitney U-tests, exploratory t-tests, and an exploratory chi-square test were performed (depending on the normal distribution); accordingly, no strategy for multiple testing was used. We considered two-sided *P* values ≤ 0.05 to be statistically significant. Differences in medians are reported using the Hodges–Lehmann estimator.

All statistical tests were performed using SPSS Statistics (IBM SPSS Statistics for Windows, Version 27.0, IBM Corp.), and all graphic representations were performed using GraphPad Prism (GraphPad Prism for Windows, Version 9.4.1. GraphPad Software). There was no need to follow up with the patients.

## Results

**Fig. 1:**
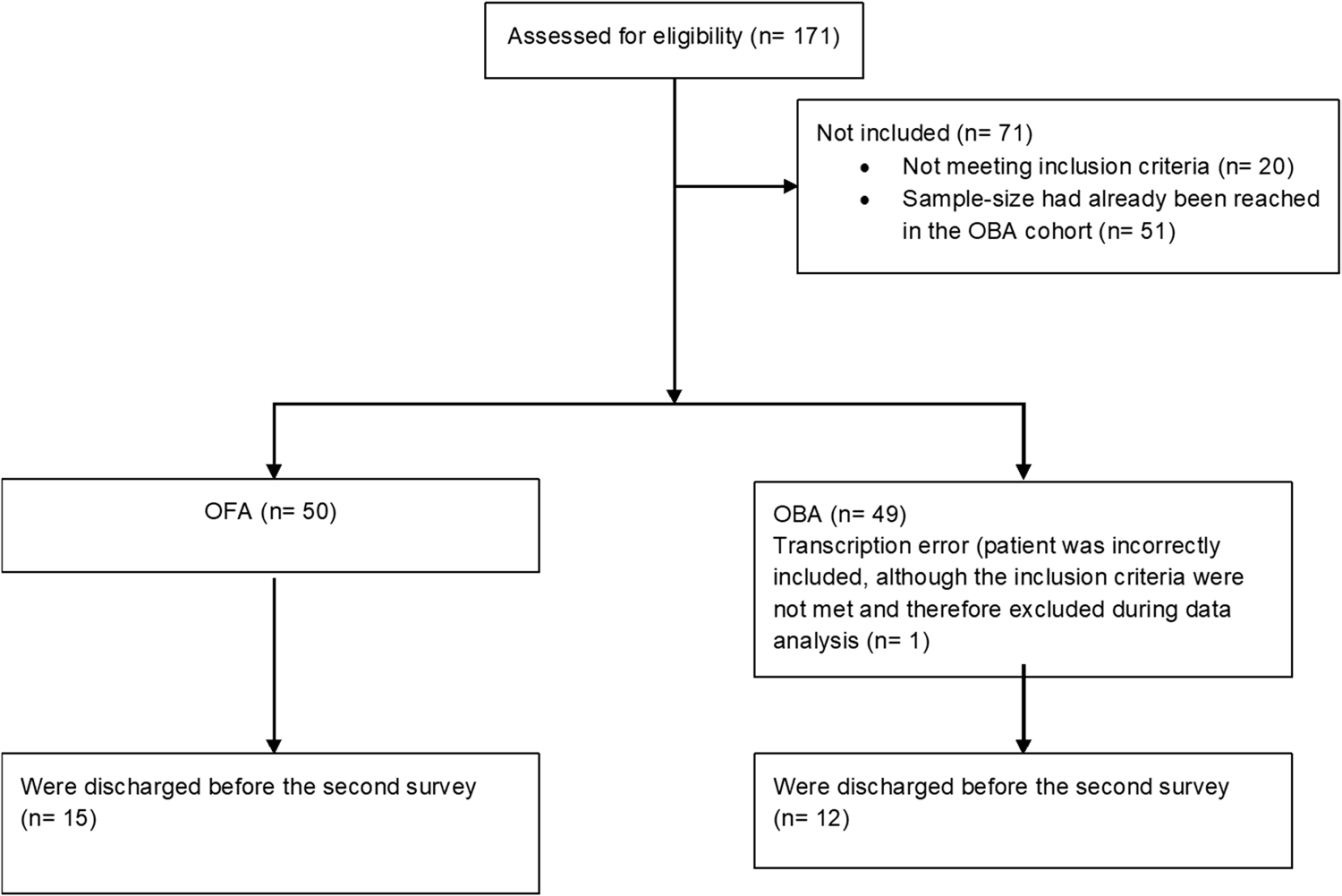
Consort flowchart. *OFA*  opioid-free anesthesia, *OBA* opioid-based anesthesia

During the investigation period, 171 patients were screened, 99 of whom were included and analyzed in this study: 50 patients in the OFA cohort and 49 patients in the OBA cohort. The flow chart is presented in Fig. 1. One patient in the OBA cohort was incorrectly included due to a transcription error and was therefore excluded from the data analysis. The investigation period was from October 2020 to July 2021. The morphometric and baseline parameters of the study participants are presented in Table 1.

**Table 1:** Subject characteristics and morphometric data

Characteristics	OFA (*N* = 50)	OBA (*N* = 49)	*P* value
Sex (f/m)	35/15	37/12	0.538^a^
Age (years), mean (± SD)	43 (10.4)	40 (13.6)	0.220^b^
Height (cm), mean (± SD)	169 (10.6)	167 (9.2)	0.835^b^
Weight (kg), mean (± SD)	130 (24.1)	127 (20.3)	0.408^b^
BMI (kg/m2), median [IQR]	44.6 [40.8–49]	44.1 [41.8–48.6]	0.994^c^
Intervention			0.384^a^
Y-roux gastric bypass (%)	12 (24%)	16 (33%)	
Omega-loop gastric bypass (%)	20 (40%)	14 (29%)	
Sleeve gastrectomy (%)	6 (12%)	10 (20%)	
SADI-S (%)	12 (24%)	9 (18%)	

*OFA* opioid-free anesthesia, *OBA* opioid-based anesthesia, *BMI* Body mass index, *SADI-S* single-anastomosis duodeno-ileal bypass with sleeve

^a^Chi-square test

^b^T-test

^c^Mann–Whitney U test

### Primary Outcome

**Fig. 2:**
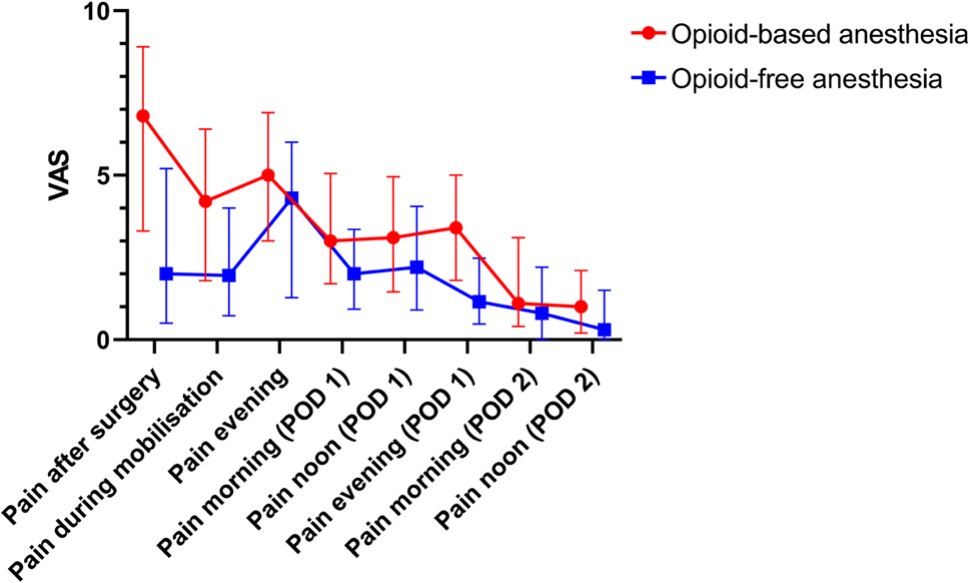
Course of postoperative pain in both cohorts within 48 h postoperatively. *VAS* visual analogue scale, *POD* postoperative day

**Fig. 3:**
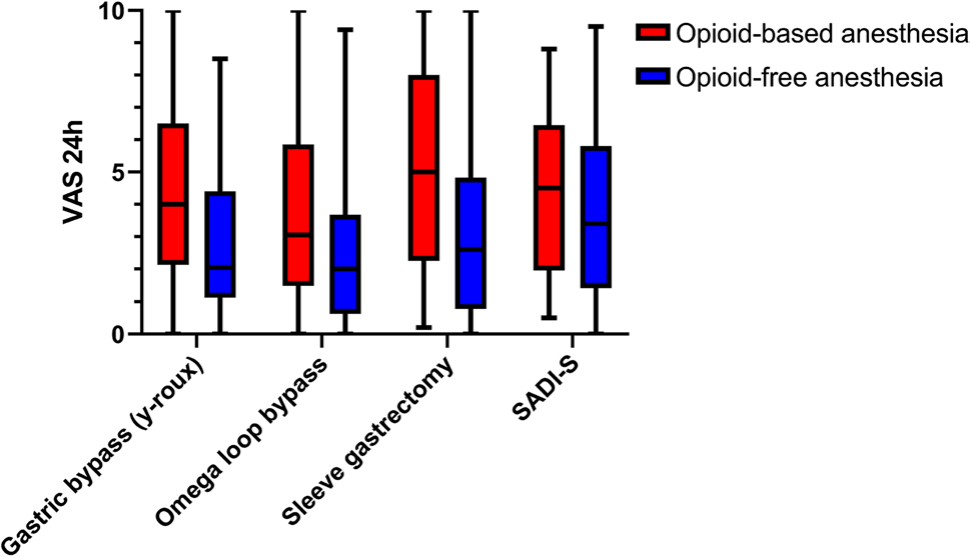
Postoperative pain within 24 h for the different types of surgery. *VAS* visual analogue scale, *SADI-S* Single anastomosis duodeno-ileal bypass with sleeve

The median [IQR] VAS in the first 24 h postoperatively was 2.2 [1–4.4] in the OFA cohort and 4.1 [2–6.5] in the OBA cohort (Hodges–Lehmann estimator of the difference, -1.5; 95.0% CI, -2 to -1; Mann–Whitney U-Test *P* ≤ 0.001). The course of postoperative pain in both cohorts over 48 h is presented in Fig. 2. Figure 3 shows the postoperative pain within 24 h for the different types of surgery.

### Secondary Outcomes

Numeric secondary outcome parameters are shown in Table 2. A total of 26 (52%) patients in the OFA cohort received continuous infusion of s-ketamine, dexmedetomidine, and lidocaine in the recovery room, whereas 15 (30%) patients received neither piritramide nor a continuous infusion of s-ketamine, dexmedetomidine, and lidocaine. We observed that 9 (18%) patients in the OBA cohort did not require piritramide. Postoperative opioid consumption (Piritramide) was lower in the OFA group (OFA: 0 [0–3.4], OBA: 6[3–9]; *P* ≤ 0,001). 26 (52%) of 50 patients in the OFA group and 38 (77.6%) of 49 patients in the OBA group received droperidol in the PACU (chi-square test, *P* = 0.008).

**Table 2:** Numeric secondary outcome parameter

Outcome	OFA	OBA	Difference in means/medians (95% CI)	*P* value
VAS 48 h median [IQR] OFA: *N* = 35; OBA: *N* = 37	1.9 [0.4–4.1]	3.1 [1.4–5.8]	−1 (−1.5 to −0.6)^a^	≤ 0.001^b^
QoR-40 24 h mean (± SD) OFA: *N* = 50; OBA: *N* = 49	166 (13.5)	155 (16.5)	11 (5 to 17.1)	≤ 0.001^c^
QoR-40 48 h mean (± SD) OFA: *N* = 35; OBA: *N* = 37	183 (9.7)	173 (11.6)	10 (5 to 15)	≤ 0.001^c^
PONV (3 (worst) – 15 (best)) median [IQR] OFA: *N* = 50; OBA: *N* = 49	13 [11–15]	10 [6.5–13]	2 (1 to 4)^a^	≤ 0.001^b^
Postoperative opioid requirements (Piritramide) 24 h (mg) median [IQR] OFA: *N* = 50; OBA: *N* = 49	0 [0–3.4]	6 [3–9]	−3 (−6 to −3)^a^	≤ 0.001^b^

Values are mean (SD) and median [IQR 25-75^th^ percentile]

*OFA* opioid-free anesthesia, *OBA* opioid-based anesthesia, *VAS* visual analogue scale, *QoR-40* Quality of recovery 40 questionnaire

^a^Hodges–Lehmann estimator

^b^Mann–Whitney U test

^c^T-test

We found statistically significant differences regarding QoR-40 scores after 24 h (OFA: 166 (13.5), OBA: 155 (16.5); *P* ≤ 0.001) and 48 h (OFA: 183 (9.7), OBA: 173 (11.6); ≤ 0.001) in favor of the OFA group. The differences in QoR-40 regarding postoperative pain are shown in Table 3. The median LOS was 4 days [3–4] and 4 days [3–4] in the OFA and OBA groups, respectively (Mann–Whitney U Test, *P* = 0,609). In the OFA cohort, 15 (30%) patients were discharged between 24 and 48 h after surgery, and 12 (24%) patients were discharged between 24 and 48 h in the OBA cohort (chi-square test, *P* = 0.538).

**Table 3:** Differences in QoR-40 in regard to postoperative pain

	*N*	QoR-40
OFA mean VAS 24 h > 3 (± SD)	22	160.1 (12.7)
OFA mean VAS 24 h < 3 (± SD)	28	170.8 (12.3)
*P* value		0.004^a^
OBA mean VAS 24 h > 3 (± SD)	35	151.3 (16.7)
OBA mean VAS 24 h < 3 (± SD)	14	164.4 (12.2)
*P* value		0,011^a^

Values are mean (SD)

*OFA* opioid-free anesthesia, *OBA* opioid-based anesthesia, *QoR-40* Quality of recovery 40 questionnaire

^a^T-test

## Discussion

The use of opiate-free anesthesia is reported to be associated with less postoperative pain and better postoperative recovery in bariatric surgery, but there is no universally accepted concept yet, and some aspects have not been adequately investigated. In this prospective cohort study comparing an OFA regimen with an OBA regimen in patients undergoing bariatric surgery, patients were surveyed 24 and 48 h postoperatively regarding their postoperative pain, as well as their postoperative recovery, to investigate whether the results found in a controlled trial setting can be confirmed in everyday clinical practice. Our hypothesis was that the results found in controlled trials, could be transferred to everyday clinical practice without a controlled trial setting and that, accordingly, patients receiving OFA would have less postoperative pain and better postoperative recovery. We observed statistically significant differences in postoperative pain and postoperative recovery between the cohorts over a period of 48 h postoperatively. Patients in the OFA cohort had significantly lower pain scores, lower opioid requirements, and recovered faster than patients in the OBA cohort.

Given that patients undergoing bariatric surgery have high rates of PONV [16, 17] and a higher risk of postoperative respiratory depression and sleep apnea [18, 25], the investigation of OFA for bariatric surgery is scientifically and clinically relevant. Despite ongoing controversy regarding the beneficial effects of OFA concepts [13], our results are comparable to those of smaller randomized controlled studies [1–3, 8] and previous results [10]. Our study not only confirms these results but also demonstrates that the beneficial effects of OFA for bariatric surgery can be achieved in everyday clinical practice and not only in a controlled trial setting. Our results are also in line with the findings of Urvoy et al., who conducted a partly similar observational study but included patients receiving total hip arthroplasty [9].

We could show a statistically significant difference regarding PONV within 24 h postoperatively in favor of the OFA group. These findings are in line with the results of previous investigations [10, 14] and confirm that the application of OFA leads to a reduction of PONV in patients receiving bariatric surgery in everyday clinical practice. The relatively large difference in postoperative opioid requirements observed in our study might be partly due to the intraoperative s-ketamine, dexmedetomidine, and lidocaine infusion being continued in the recovery room. Consequently, opioid administration was the second choice for treating acute postoperative pain in the OFA group. Therefore, whether strict intraoperative use of OFA (without continuing into the postoperative period) would have led to a similar reduction in postoperative opioid administration remains unclear.

We assumed, from clinical experience and due to the fact that these parameters are included in the QoR-40, that the differences found in the QoR-40 were related to the outcome parameters of postoperative pain, PONV, and postoperative opioid consumption. This would also correspond to the findings presented in Table 3. Accordingly, we can conclude that the application of OFA leads to an improved patient experience due to the improvement of several parameters that influence postoperative recovery.

According to a retrospective study by Mulier et al., OFA improves the outcome of bariatric procedures; thus, this regimen can be assumed to be safe for use in this patient population [24]. Our study was not designed to specifically investigate the safety of OFA (e.g. the occurrence of bradycardia, as described by Beloeil et al. in 2021 [15]). We are therefore unable to draw definite conclusions regarding the safety of OFA, but we are currently performing a retrospective evaluation of this issue at our center.

We found no difference regarding LOS, even though the patients in the OFA group showed statistically significant differences in the QoR-40 questionnaire 24 and 48 h postoperatively. These findings contradict the results of a comprehensive retrospective analysis of a large patient collective done by Mulier et al. [24], which showed that patients receiving OFA had a shorter LOS, but are in line with the findings of a recent meta-analysis, even though only two studies were included for this parameter in the analysis [10]. However, it must be taken into account that LOS is not solely dependent on the anesthesiologic procedure and can be influenced by a variety of other factors (e.g. different health care systems, center-specific SOPs). Compared with other studies, our patients had a longer LOS (4 days vs. 3 days) [24, 26]. We suspect that this difference may be due to the fact that in our center, patients are admitted the day before surgery.

A limitation of this study is that it was conducted at a single center, limiting the generalizability of our results. Due to the size of our department (approximately 180 anesthetists), we nevertheless believe that these methods can be applied by a large variety of anesthetists.

## Conclusion

The results of this study showed that the introduction of a concept for OFA in clinical routine practice was associated with less postoperative pain, lower opioid requirements and less PONV and patients demonstrated improved postoperative recovery. Further studies are needed to determine the best anesthetic protocol and to investigate safety aspects with this form of anesthesia.

## Supplementary Information

Below is the link to the electronic supplementary material.

Supplementary file1 (PDF 106 KB)

Supplementary file2 (PDF 121 KB)

## Data Availability

The data will be made available on justified request to the corresponding author.
