# Supplementary material for: The Performance of Opioid-Free Anesthesia for Bariatric Surgery in Clinical Practice
Source: Obes Surg. 2023 Apr 27;33(6):1687–93. doi: 10.1007/s11695-023-06584-5 (PMC10234923; doi:10.1007/s11695-023-06584-5)
Supplement: Supplementary file 1 — Supplementary file1 (PDF 106 KB) [file 11695_2023_6584_MOESM1_ESM.pdf]

Opioid-based anesthesia for bariatric surgery

Anesthesia induction

Induce anesthesia with 150-300 µg Fentanyl, 200-250 mg Propofol and 100 mg Rocuronium

Maintenance of anesthesia and intraoperative medication

- Maintain anesthesia with a continuous infusion of remifentanil (0,1-0,5µg/kg ideal BW/min)
- Adjust Desflurane/Sevoflurane to achieve a MAC of approximately 0.5 to 1
- Maintain deep paralysis with rocuronium (aim for PTC <8)
- 2-4 g Magnesiumsulfate as short infusion
- 2 g Metamizol iv
- 40 mg Parecoxib oder 75mg Diclofenac iv
- 8 mg Ondansetron iv
- 8 mg Dexamethason iv
- Consider 2.5 mg Droperidol iv

End of anesthesia

- Stop remifentanil infusion when gastroscopy is performed
- Pain therapy: piritramide iv
- Stop Desflurane/Sevoflurane and wash out a high flow when gastroscopy is performed
- Antagonize muscle paralysis with sugammadex (based on total body weight) at skin suture

Recovery area

- Pain therapy: piritramide iv
- Mobilize after 1h
