# Supplementary material for: The Performance of Opioid-Free Anesthesia for Bariatric Surgery in Clinical Practice
Source: Obes Surg. 2023 Apr 27;33(6):1687–93. doi: 10.1007/s11695-023-06584-5 (PMC10234923; doi:10.1007/s11695-023-06584-5)
Supplement: Supplementary file 2 — Supplementary file2 (PDF 121 KB) [file 11695_2023_6584_MOESM2_ESM.pdf]

# Opioid-free anesthesia for bariatric surgery

## Anesthesia induction

Add the following medication to 1 syringe (for syringe pump)

- Dexmedetomidine 400 µg (4ml)
- S-Ketamine 50 mg (2ml-10ml depending on the concentration)
- Lidocaine 400 mg (20ml)
- Add NaCl 0.9% to achieve a volume of 40 ml in total

The mixture contains the following concentrations

- Dexmedetomidine 10 µg/ml
- S-Ketamine 1.25 mg/ml
- Lidocaine 10 mg/ml

Start infusion when patient is on the OR table at 20ml/h (s-ketamine: 25 mg/h; dexmedetomidine: 200 µg/h; lidocaine: 200 mg/h)

Induce anesthesia with 200-250 mg Propofol and 100 mg Rocuronium

## Maintenance of anesthesia and intraoperative medication

- Reduce infusion (dexmedetomidine/s-ketamine/lidocaine) to 5-10ml/h depending on hemodynamics (s-ketamine: 6.25-12.5 mg/h; dexmedetomidine 50-100 µg/h; lidocaine 50-100 mg/h)
- Adjust Desflurane/Sevoflurane to achieve a MAC of approximately 05. to 1
- Maintain deep paralysis with rocuronium (aim for PTC <8)
- 2-4 g Magnesiumsulfate as short infusion
- 2 g Metamizole iv
- 40 mg Parecoxib oder 75 mg Diclofenac iv
- 8 mg Ondansetron iv
- 8 mg Dexamethason iv
- Consider 2.5g mg Droperidol iv

## End of anesthesia

- Stop infusion (Dexmedetomidine/s-ketamine/lidocaine) when gastroscopy is performed
- Stop Desflurane/Sevoflurane and wash out a high flow when gastroscopy is performed
- Antagonize muscle paralysis with sugammadex (based on total body weight) at skin suture

**Recovery area**

- Re-start infusion (dexmedetomidine/s-ketamine/lidocaine) at 5ml/h if necessary for pain control
- Piritramide iv rescue pain therapy
- Mobilize after 1h
